# Relationship Between Pre- and Post-Operative C-Reactive Protein (CRP), Neutrophil-to-Lymphocyte Ratio (NLR), and Platelet-to-Lymphocyte Ratio (PLR) With Post-Operative Pain After Total Hip and Knee Arthroplasty: An Observational Study

**DOI:** 10.7759/cureus.43782

**Published:** 2023-08-20

**Authors:** Akshay Rathee, Manoj K Chaurasia, Manish K Singh, Vinita Singh, Dinesh Kaushal

**Affiliations:** 1 Anesthesiology and Critical Care, King George’s Medical University, Lucknow, IND; 2 Anesthesiology and Critical Care, King George's Medical University, Lucknow, IND

**Keywords:** total hip and knee arthroplasty, post-operative pain, platelet to lymphocyte ratio (plr), neutrophil to lymphocyte ratio (nlr), c-reactive protein (crp)

## Abstract

Background: Anesthetic technique and postoperative pain management are crucial for total joint arthroplasty (TJA) patients. The neutrophil-to-lymphocyte ratio (NLR), platelet-to-lymphocyte ratio (PLR), and C-reactive protein (CRP) are new, simple, and cost-effective predictors for prognosis. The predictive value of NLR as an inflammatory marker can predict post-operative pain caused by inflammatory pathways secondary to surgical trauma. CRP is also the most sensitive and specific biomarker of inflammation whereas PLR was also recently considered a possible marker for inflammation which may further contribute to pain and sequelae. Thus, anesthetists can make decisions about the amount, time, and type of analgesic to use based on preoperative values of these parameters to provide maximum postoperative pain control and facilitate early rehabilitation. Thus, the current study was conducted to determine the relationship between CRP, NLR, and PLR levels and the intensity of pain in patients following total hip arthroplasty (THA) and total knee arthroplasty (TKA).

Materials and Methods: A total of 105 patients scheduled for THA and TKA fulfilling the study's inclusion criteria were enrolled. Inclusion criteria of the study were all the patients giving written consent, ASA Grade I-III, patients between 18 and 90 years who were scheduled for elective lower extremity TJA, and all the patients who remained admitted until stitches were removed. Patients were given intrathecal 15 mg hyperbaric bupivacaine via 25G atraumatic spinal needle in the L3-L4 interspace. The recorded data were demographic characteristics, preexisting comorbidities, number of blood transfusions, and operation time, postoperative analgesics given, duration of hospital stay, time of mobility, pain scoring as per visual analog scale (VAS) scoring system with an aim to establish a relationship between pre- and post-operative (Days 3 & 5) CRP, NLR, and PLR with post-operative pain after THA and TKA.

Result: The present study demonstrated a significant correlation (p < 0.002) between preoperative and postoperative NLR with pain after TJA whereas PLR and CRP did not show any significant relationship with post-operative pain after THA and TKA. A significantly higher NLR ratio was observed for patients on all the periods of observation (pre-op., Day 3, and Day 5). Pre-op. and Day 5 NLR of patients who required transfusion were significantly higher than those who did not require transfusion and patients with higher NLR values could be mobilized significantly later and had significantly higher duration of hospital stay. The correlation of CRP levels and PLR levels at different time intervals did not show a significant correlation with Day 3 and Day 5 pain scores.

Conclusion: The present study demonstrated a significant correlation between preoperative and postoperative NLR with pain after TJA.

## Introduction

Osteoarthritis (OA) is one of the most prevalent chronic degenerative diseases of the middle-aged and elderly. Worldwide, it is believed that between 10% and 15% of persons over the age of 60 suffer from OA. Around the world, roughly 86.7 million people are anticipated to have symptomatic knee OA, according to the 2020 report [1]. Total joint arthroplasty (TJA), which includes total knee arthroplasty (TKA) and total hip arthroplasty (THA), has emerged as the most successful technique for restoring physical function and alleviating joint pain in patients with end-stage hip or knee arthritis, respectively [2].

Although TJA helps to reduce pain in the majority of patients, a variety of postoperative complications may result in adverse outcomes, including periprosthetic joint infection (PJI), one of the most serious complications of TJA, which occurs in approximately 1%-3% of all TKAs, and moderate to severe persistent postoperative pain (PPP) in the operated knee three months after surgery, which is experienced by approximately 1%-3% of all TKAs [3].

The C-reactive protein (CRP) levels revert to preoperative levels within three weeks; however, they may be raised for a variety of reasons in addition to postoperative infection, given the high prevalence of TJA in elderly patients with concomitant diseases [4]. Recently, the neutrophil-to-lymphocyte ratio (NLR) and platelet-to-lymphocyte ratio (PLR) has been examined and proven to be useful in predicting the outcome or prognosis of a variety of disorders, including oncological, inflammatory, and some infectious diseases [5]. CRP is also the most sensitive and specific biomarker of inflammation and necrosis in the laboratory. The serum CRP level is frequently used as a screening test for infection due to its simplicity, affordability, and sensitivity [6]. CRP levels in the blood increase 10-fold to 100-fold during inflammation. It has been established that CRP increases dramatically in the first three days following surgery, including the uncomplicated post-operative period [7-8]. According to Kim et al., only 24% of cases of CRP elevation in the post-operative period following large joint arthroplasty are due to the development of intra-articular infection, while 56% are due to its response to the development of an infectious process occurring outside the joint [9].

According to Forget et al., the normal NLR value in healthy people should be 1.65, but within a larger range of 0.78-3.53. However, the average NLR value has been found to be greater (2.15), and it varies with race [10-11]. The predictive value of NLR as an inflammatory marker in a variety of diseases can predict the correlation with postoperative pain caused by inflammatory pathways secondary to surgical trauma and/or surgery-related direct nerve trauma. Thus, anesthetists can make decisions about the amount, time, and type of analgesic to use based on preoperative NLR readings [12]. The statement states that based upon the preoperative NLR, the anesthetists can be more vigilant regarding the management of post-operative pain regarding the choice of drugs, amount, and time the analgesics are to be used.

Platelets activated in response to the rupture of atherosclerotic plaques or endothelial cell erosion promote the production of thrombus, hence promoting atherothrombotic disease [13]. PLR was recently considered a possible marker for inflammation in a variety of illnesses, including rheumatoid arthritis, systemic lupus erythematosus, and epithelial ovarian carcinoma [14].

Determining the level of CRP in the postoperative period following TJA can be used to measure the pain syndrome, allowing for an objective assessment and adjustment of the anesthetic strategy used. The NLR and PLR are simple and inexpensive biomarkers for systemic inflammation. To provide maximum post-operative pain control and to facilitate early rehabilitation in THR and TKR patients, a screening test to rule out the risk of infection is critical. Thus, the current study was conducted to determine the relationship between CRP, NLR, and PLR levels and the intensity of pain in patients following TKA and THA.

## Materials and methods

After getting clearance from the ethical committee, the study was conducted in the Department of Anesthesiology and Critical Care, King George’s Medical University with CTRI/2021/02/031240. This prospective, observational, cohort study was conducted for one year to correlate post-operative pain of patients of THA and TKA with pre- and post-operative CRP, NLR, and PLR. A total of 105 patients scheduled for THA and TKA fulfilling the inclusion criteria of the study were enrolled in the study after obtaining informed consent. Inclusion criteria of the study were all the patients giving written consent, ASA Grade I-III, patients between 18 and 90 years who were scheduled for elective lower extremity TJA, and all the patients who remained admitted until stitches were removed. Exclusion criteria were the patients having complications of pneumonia, wound infection, urinary tract infection, malignancy, chronic inflammatory disease history, or corticosteroid use and emergency operations.

The sample size was calculated by taking the maximum area under the curve (AUC) of the parameter among all the parameters under study using the formula [15]:

$n = \frac{z_{\alpha }^{2 }S_{n}\left ( 100 - S_{_{n}} \right )}{pe^{2}}$

Where,

$S_{n}$ = 0.613 (61.3%), AUC of NLR (having maximum AUC among the parameters under study)

p = 0.155 (15.5%), prevalence of disease as found in the study 

e = 0.25, error factor for detecting results with 90% power of study Type I error (level of significance) α=0.05

Data loss factor = 10%

Then, the minimum sample size required would be n = 105.

Procedure

Patients were given intrathecal 15 mg hyperbaric bupivacaine via 25G atraumatic spinal needle in the L3-L4 interspace in a sitting position. Preoperative periarticular or intraarticular injections were not given. Patients were mobilized and a standardized rehabilitation protocol was started on the first day after surgery in both groups. Patients with preoperative CRP levels > 10 mg/L were also excluded, as well as any other inflammatory or medical complication during the hospital stay.

All patients received the same prophylactic antibiotics (1 g of ceftriaxone) within 1 h before incision. IV antibiotics were administered to all patients for 2 days. Tolerable weight-bearing ambulation was allowed on the second postoperative day after drain removal. Rescue analgesics were given in form of IV Tramadol and transdermal patches (buprenorphine and fentanyl patches). The recorded data were demographic characteristics, preexisting comorbidities, number of blood transfusions, operation time, postoperative analgesics given, duration of hospital stay, and pain scoring as per the VAS scoring system.

We routinely evaluated preoperative white blood corpuscles (%), CRP, and platelet count. Venous blood samples of all patients were obtained preoperative 24 h before surgery, postoperative on Day 3 and Day 5. The VAS scores were noted on Day 3 and Day 5 and were denoted from a scale of 0-10 where scores 1-3 = low pain, 4-6 = moderate pain, and a score of >6 = high pain. We also noted the day of mobilization, duration of hospital stay, and post-operative complications.

Statistical analysis

The statistical analysis was done using Statistical Package for Social Sciences (SPSS) Version 21.0 Statistical Analysis Software (IBM Corp., Armonk, NY). The values were represented in number (%) and mean ± standard deviation (SD). Student’s t-test, Pearson’s correlation, Spearman’s correlation, and analysis of variance (ANOVA) were applied for data analysis as applicable. A p-value < 0.05 was considered statistically significant.

## Results

Table 1 shows the demographic profile of the study population. The age of patients enrolled in the study ranged between 24 and 84 years, mean age was 50.02 ± 10.70 years. The majority of the patients were aged 41-60 years (71.4%). Male preponderance was observed (67.5%) in the study population.

**Table 1:** Demographic profile of the study population (N=105). SD, standard deviation

SN	Characteristics	No. of patients	Percentage
1	Age		
	≤40 years	16	15.2
	41-60 years	75	71.4
	>60 years	14	13.3
	Mean age ± SD (Range)	50.02 ± 10.70 (24-84)	
2	Gender		
	Female	34	32.4
	Male	71	67.5

Table 2 shows the clinical profile of the study population. The majority of the patients enrolled in the study were ASA Grade I & II (94.3%), and only six (5.7%) patients belonged to ASA Grade III. Out of 105 patients enrolled in the study, 37 (35.2%) had reported comorbidities. The most common comorbidity was diabetes (13.3%) followed by hypertension (8.6%). Both diabetes and hypertension (7.6%), chronic obstructive pulmonary disease (COPD), history of (H/o) myocardial infarction, and H/o transient ischemic attack (TIA) were reported by 1.9%, 2.9%, and 1.0% of patients, respectively.

**Table 2:** Clinical profile of study population (N=105). TIA, transient ischemic attack; COPD, chronic obstructive pulmonary disease

SN	Characteristics	No. of patients	Percentage
1	ASA Grade		
	Grade I	47	44.8
	Grade II	52	49.5
	Grade III	6	5.7
2	Comorbidities	37 (35.2%)	
	Diabetes	14	13.3
	Hypertension	9	8.6
	Hypertension + Diabetes	8	7.6
	COPD	2	1.9
	H/o Myocardial infarction	3	2.9
	H/o TIA	1	1.0
3	Transfusion required	20	19.0
4	Diagnosis		
	Arthritis other than osteoarthritis	32	30.5
	Osteoarthritis (knee/hip)	73	69.5

The mean VAS score for pain on Day 3 was 4.30 ± 1.32 (Range 1-6), two thirds of patients had moderate pain (score 4-6). On Day 5 mean pain score was 3.30 ± 0.96 (Range 2-6); the majority of patients had a low level of pain (score 1-3). A statistically significant decline in Day 3 pain was observed on Day 5 (0.99 ± 1.24). The percentage decline in pain on Day 5 was 23.0%. A significant correlation (p<0.002) between pain at Day 3 and Day 5 was found, and the level of correlation was found to be mild (r=0.449) (Table 3).

**Table 3:** Severity of post-operative pain on Day 3 and Day 5. VAS, visual analog scale; SD, standard deviation

SN	Severity of post-operative pain	Day 3		Day 5	
		No.	%	No.	%
1	Low pain (VAS 1-3)	35	33.3	59	56.2
2	Moderate pain (VAS 4-6)	70	66.7	46	43.8
	Mean pain ± SD (Median; Range)	4.30 ± 1.32 (4; 1-6)		3.30 ± 0.96 (3; 2-6)	
	Mean change in the severity of pain on Day 5			0.99 ± 1.24 (23.0%)	
	Wilcoxon signed rank test			Z=-6.481; p<0.001	

Cases with low Day 3 pain and moderate pain had comparable levels of CRP preoperatively as well as on Day 3 and Day 5 (Table 4).

**Table 4:** Association of Day 3 pain with CRP levels at different time intervals. CRP, C-reactive protein; SD, standard deviation

SN	Time	Low pain (n=35)		Moderate pain (n=70)		Student's ‘t’ test	
		Mean	SD	Mean	SD	‘t’	‘p’
1	Pre-op.	3.12	1.61	3.67	1.54	-1.676	0.097
2	Day 3	3.95	1.71	3.86	1.60	0.266	0.791
3	Day 5	3.30	1.28	3.46	1.58	-0.519	0.605

Cases with low Day 5 pain and moderate pain had comparable levels of CRP preoperatively as well as on Day 3 and Day 5 (Table 5).

**Table 5:** Association of CRP and pain on Day 5. CRP, C-reactive protein; SD, standard deviation

SN	Time	Low pain (n=59)		Moderate pain (n=46)		Student's ‘t’ test	
		Mean	SD	Mean	SD	‘t’	‘p’
1	Pre-op.	3.33	1.50	3.69	1.66	-1.166	0.246
2	Day 3	3.93	1.77	3.84	1.45	0.261	0.794
3	Day 5	3.27	1.48	3.59	1.48	-1.119	0.266

The correlation of CRP levels at different time intervals did not show a significant correlation with Day 3 and Day 5 pain scores (Table 6).

**Table 6:** Correlation of Day 3 and Day 5 pain with CRP at different time intervals (Spearman’s correlation). CRP, C-reactive protein; VAS, visual analog scale

SN	CRP level	Day 3 VAS		Day 5 VAS	
		‘r’	‘p’	‘r’	‘p’
1	Pre-op.	0.136	0.168	0.071	0.474
2	Day 3	-0.066	0.504	0.013	0.893
3	Day 5	0.079	0.422	0.140	0.155

A significantly higher NLR ratio was observed for patients on all the periods of observation (pre-op., Day 3, and Day 5) with a higher level of Day 3 pain (moderate level) as compared to a lower level of pain (low pain) (Table 7).

**Table 7:** Association of NLR and pain on Day 3. NLR, neutrophil-to-lymphocyte ratio; SD, standard deviation

SN	Time	Low pain (n=35)		Moderate pain (n=70)		Student's ‘t’ test	
		Mean	SD	Mean	SD	‘t’	‘p’
1	Pre-op.	2.65	0.65	3.19	1.25	-2.413	0.018
2	Day 3	3.14	0.51	3.79	1.05	-3.481	0.001
3	Day 5	4.01	0.88	4.56	1.07	-2.648	0.009

A higher NLR ratio was observed for patients on all the periods of observation (pre-op., Day 3, and Day 5) with a higher level of Day 5 pain (moderate level) as compared to a lower level of pain (low pain). Pre-operative NLR of patients with low and moderate pain were found to be comparable (Table 8).

**Table 8:** Association of NLR and pain on Day 5. NLR, neutrophil-to-lymphocyte ratio; SD, standard deviation

SN	Time	Low pain (n=59)		Moderate pain (n=46)		Student's ‘t’ test	
		Mean	SD	Mean	SD	‘t’	‘p’
1	Pre-op.	2.87	0.95	3.19	1.28	-1.461	0.147
2	Day 3	3.37	0.77	3.84	1.10	-2.537	0.013
3	Day 5	4.11	0.92	4.71	1.10	-3.021	0.003

Significant correlations between Day 3 and Day 5 pain were observed with pre-op., Day 3, and Day 5 NLR (Table 9).

**Table 9:** Correlation of Day 3 and Day 5 pain with NLR at different time intervals (Spearman’s correlation). NLR, neutrophil-to-lymphocyte ratio; VAS, visual analog scale

SN	NLR	Day 3 VAS		Day 5 VAS	
		‘r’	‘p’	‘r’	‘p’
1	Pre-op.	0.236	0.015	0.192	0.049
2	Day 3	0.325	0.001	0.267	0.006
3	Day 5	0.372	<0.001	0.315	0.001

Pre-operative and Day 3 PLR of patients with low Day 3 pain and moderate pain were comparable. The Day 5 PLR of patients with moderate Day 3 pain was significantly higher as compared to those with low Day 3 pain (Table 10).

**Table 10:** Association of PLR and pain on Day 3. PLR, platelet-to-lymphocyte ratio; SD, standard deviation

SN	Time	Low pain (n=35)		Moderate pain (n=70)		Student's ‘t’ test	
		Mean	SD	Mean	SD	‘t’	‘p’
1	Pre-op.	73.68	23.40	78.88	19.88	-1.191	0.236
2	Day 3	77.98	30.46	73.89	18.28	0.858	0.393
3	Day 5	74.64	14.31	82.52	29.63	-2.025	0.045

Pre-operative and Day 5 PLR of patients with low Day 5 pain and moderate pain were comparable. Day 3 PLR of patients with low Day 3 pain was significantly higher as compared to those with moderate Day 3 pain (Table 11).

**Table 11:** Association of PLR and pain on Day 5. PLR, platelet-to-lymphocyte ratio

SN	Time	Low pain (n=59)		Moderate pain (n=46)		Student's ‘t’ test	
		Mean	SD	Mean	SD	‘t’	‘p’
1	Pre-op.	75.55	17.44	79.20	25.19	-0.877	0.382
2	Day 3	79.49	26.78	69.82	15.63	2.176	0.032
3	Day 5	81.54	20.94	77.78	16.31	1.005	0.317

Pre-op., Day 3, and Day 5 PLR did not show any significant correlation with post-operative pain at different time intervals (Table 12).

**Table 12:** Correlation of Day 3 and Day 5 pain with PLR at different time intervals (Pearson’s correlation). PLR, platelet-to-lymphocyte ratio; VAS, visual analog scale

SN	PLR	Day 3 VAS		Day 5 VAS	
		‘r’	‘p’	‘r’	‘p’
1	Pre-op.	0.119	0.227	0.188	0.055
2	Day 3	-0.181	0.065	-0.162	0.098
3	Day 5	0.165	0.093	0.047	0.635

Patients with Day 3 moderate pain as compared to those with low pain could be mobilized significantly later (2.40 ± 0.49 vs. 2.03 ± 0.17 days) and had a significantly higher duration of hospital stay (11.83 ± 2.01 vs. 10.00 ± 0.00 days) (Table 13).

**Table 13:** Association of outcome parameters and pain on Day 3. SD, standard deviation

SN	Time	Low pain (n=35)		Moderate pain (n=70)		Student's ‘t’ test	
		Mean	SD	Mean	SD	‘t’	‘p’
1	Time of mobility	2.03	0.17	2.40	0.49	-4.319	<0.001
2	Hospital stay	10.00	0.00	11.83	2.01	-5.377	<0.001

Patients with Day 5 moderate pain as compared to those with low pain could be mobilized significantly later (2.43 ± 0.50 vs. 2.15 ± 0.36 days) and had a significantly higher duration of hospital stay (11.83 ± 2.01 vs. 10.75 ± 1.57 days) (Table 14).

**Table 14:** Association of outcome parameters and pain on Day 5. SD, standard deviation

SN	Time	Low pain (n=59)		Moderate pain (n=46)		Student's ‘t’ test	
		Mean	SD	Mean	SD	‘t’	‘p’
1	Time of mobility	2.15	0.36	2.43	0.50	–3.347	0.001
2	Hospital stay in days	10.75	1.57	11.83	2.01	–3.088	0.003

Table 15 shows the association of NLR (Pre-op., Day 3, and Day 5) with demographic variables. At all the periods of observation (Pre-op., Day 3, and Day 5) NLR was maximum for patients aged >60 years followed by patients aged 41-60 years, and minimum for patients aged ≤40 years. But significant differences were observed for Pre-op. values only, i.e. pre-operative NLR of patients aged >60 years was found to be significantly higher than those aged ≤40 years and 41-60 years. At all the periods of observation (Pre-op., Day 3, and Day 5) differences in NLR of male and female patients were not found to be significant statistically.

**Table 15:** Association of NLR and demographic variables. NLR, neutrophil-to-lymphocyte ratio; ANOVA, analysis of variance

SN	Variable	No.	Pre-op.		Day 3		Day 5	
			Mean	SD	Mean	SD	Mean	SD
1	Age Group							
	≤40 years	16	2.35	0.47	3.28	0.74	4.08	1.17
	41-60 years	75	3.07	1.03	3.56	0.88	4.42	0.94
	>60 years	14	3.44	1.69	4.00	1.38	4.48	1.39
	ANOVA		F=4.239; p=0.017		F=2.240; p=0.112		F=0.766; p=0.468	
2	Gender							
	Female	34	3.02	1.17	3.54	0.94	4.34	1.05
	Male	71	3.01	1.09	3.59	0.97	4.39	1.04
	Student's ‘t’ test		‘t’=0.034; p=0.973		‘t’=-0.285; p=0.776		‘t’=-0.232; p=0.817	

Table 16 shows the association between NLR and transfusion requirements of the patients. Pre-op. and Day 5 NLR of patients who required transfusion were significantly higher than those who did not require transfusion. Day 3 NLR of patients requiring transfusion was higher than those who did not require transfusion but the difference was not found to be significant statistically.

**Table 16:** Association of NLR and transfusion requirements. NLR, neutrophil-to-lymphocyte ratio; SD, standard deviation

SN	Transfusion requirement	No.	Pre-op.		Day 3		Day 5	
			Mean	SD	Mean	SD	Mean	SD
1	Not required	85	2.73	0.97	3.50	0.93	4.26	1.03
2	Transfusion required	20	4.19	0.92	3.88	1.01	4.87	0.95
	Student's ‘t’ test		‘t’=-6.095; p<0.001		‘t’=-1.610; p=0.111		‘t’=-2.402; p=0.018	

On all the periods of observation, patients with a history of myocardial infarction and a history of TIA had higher NLR but differences with patients with other comorbidities/no comorbidity were found to be statistically significant only for Pre-op. NLR (Table 17).

**Table 17:** Association of NLR and comorbidities. H/o, history of; NLR, neutrophil-to-lymphocyte ratio; ANOVA, analysis of variance; COPD, chronic obstructive pulmonary disease; SD, standard deviation; TIA, transient ischemic attack

SN	Comorbidities	No.	Pre-op.		Day 3		Day 5	
			Mean	SD	Mean	SD	Mean	SD
1	No comorbidity	68	3.10	1.09	3.56	0.96	4.39	1.12
2	Diabetes	14	2.70	0.64	3.70	0.99	4.36	1.10
3	Hypertension	9	2.60	0.61	3.29	0.84	4.29	0.71
4	Hypertension + Diabetes	8	2.28	0.38	3.25	0.72	4.13	0.80
5	COPD	2	1.98	0.58	3.04	0.21	3.81	1.27
6	H/o Myocardial infarction	3	5.74	1.28	5.00	0.00	5.11	0.17
7	H/o TIA	1	5.00	0.00	5.00	0.00	5.30	0.00
	ANOVA		F=6.330; p<0.001		F=2.049; p=0.066		F=0.550; p=0.066	

## Discussion

The present study demonstrated a significant correlation between preoperative and postoperative NLR with pain after TJA. The correlation of CRP levels at different time intervals did not show a significant correlation with Day 3 and Day 5 pain scores. PLR at pre-operative, Day 3, and Day 5 did not show any significant correlation with post-operative pain at different time intervals. Post-operative CRP level is an indicator reflecting the invasiveness of surgical procedures. The elevated CRP levels during the postoperative period are known to be closely associated with postoperative complications including infections. Godoy et al. [16] concluded that there was no statistically significant correlation between postoperative problems and preoperative CRP and do not support routine CRP and erythrocyte sedimentation rate (ESR) investigation as part of preoperative evaluation for elective TKA and similar results were seen in our study. Kim et al. [9] demonstrated that high CRP levels following TKA can be caused by a variety of factors periprosthetic infection, cardiovascular difficulties, gastrointestinal problems, urologic problems, and respiratory problems. Sargin et al. [17] concluded that preoperative and postoperative values of CRP levels increased much more than NLR Levels. By the fifth postoperative day, the NLR had reverted to preoperative and NLR levels responded more rapidly to surgical stress than CRP levels. In our study, all 105 patients were in antibiotic coverage which might have influenced the response of inflammatory markers, hence similar results were not obtained. Londhe et al. [18] concluded that in the Indian TKR patients, the CRP values take longer time to return to normal than in their Anglo-Saxon counterparts. Due to the variable nature of raised CRP, marginal elevations in CRP can be difficult to interpret and should not be utilized as an isolated test for the clinical picture. If the levels are exceedingly high, it is beneficial for distinguishing infection from inflammation. Yu et al. [19] concluded that NLR values are more accurate than CRP and may be considered a useful parameter for the diagnosis of early PJI because it is a cheap and convenient parameter to be calculated in daily practice without extra costs. In our study, we have evaluated the correlation with pain, not the infection so this might possibly explain the different results. Although CRP is a cheap and convenient serum parameter, the overall correlation was not significant with pain and pain scores and thus we do not recommend it as a good marker for correlation with pain after TJA.

Adequate management of postoperative pain results in increased patient comfort and decreased mobilization time, systemic complications, and recovery period without disturbing memories related to surgery. Inflammation triggered by surgical trauma secondary to incision, dissection, suturing, nerve stretching, or nerve compression takes part in the mechanism of postoperative pain. Hernández et al. [20] concluded that pre-surgical factors might influence post-surgical pain in patients undergoing a knee or hip arthroplasty like female gender, low socioeconomic status, increased pain, comorbidities, low back pain, and poor functional status in patients receiving hip or knee arthroplasty. In our study, preoperative and Day 5 NLR of patients who required transfusion were significantly higher than those who did not require transfusion which explains that the patients with anemia or borderline hemoglobin have higher NLR as compared with others [21]. Fisher et al. [22] concluded that in orthogeriatric patients, high NLR on admission is an independent indicator of fracture presence, a significant risk factor, and a moderate predictor of postoperative myocardial injury, high inflammatory response/infection, and in-hospital death. In our study, the pre-operative NLR of patients aged >60 years was found to be significantly higher than those aged ≤40 years and 41-60 years. During all the periods of observation, patients with a history of myocardial infarction and a history of TIA had higher NLR but differences with patients with other comorbidities/no comorbidity were found to be statistically significant only for Pre-op. NLR. Kornilov et al. [23] concluded that patients who reported moderate/severe pain before surgery also reported higher hours spent in moderate/severe pain on postoperative days 0-3, patients who had procedures lasting longer than 90 min reported more hours of moderate/severe pain, and comparable effects were observed for pain ratings associated with exercise. Only female gender, more preoperative discomfort and anxiety, and a longer surgery length were linked with increased pain following TKA. Forget et al. [10] identified that the normal NLR values in an adult, non-geriatric, population in good health are between 0.78 and 3.53. The NLR has been proposed as a basic inflammatory response biomarker [24].

Canbolat et al. [25] concluded that NLR can be accepted as a relatively objective method for the diagnosis of postoperative pain. Pathophysiologically, inflammatory blood cells (leukocytes and neutrophils) increased in the first 24 h after the surgical stress. It indicates an increased inflammatory response provoked by surgery. In our study, pain scores were related to higher NLR values and correlated with the study. Turgut et al. [26] concluded that preoperatively measured NLR may help to predict postoperative analgesic demand in patients undergoing orthognathic surgery, and thus sufficient postoperative pain control can be achieved with various preventive treatments taken at the perioperative period such as presumptive analgesia, local anesthetic administration at the end of surgery, or early administration of analgesics. We established a significant correlation with higher values of NLR with higher pain scores indicating inflammation which in turn delays the mobilization because of pain, hence may lead to complications and prolonged duration of hospital stay.

The platelet-to-lymphocyte ratio (PLR) has emerged as an informative marker revealing shifts in platelet and lymphocyte counts due to acute inflammatory and prothrombotic states. Yao et al. [15] concluded that a significant association of perioperative NLR or PLR with acute TJA-induced DVT, NLR or PLR cannot predict TJA-induced DVT accurately. In our study preoperatively, Day 3 and Day 5 PLR did not show any significant correlation with pain after TJA. We did not correlate the PLR values with DVT or other postoperative complications.

Gorial et al. [27] concluded that PLR and NMR had no significant correlation with knee OA severity. As per the literature, PLR may be significantly higher in post-operative period due to lower lymphocyte levels. But as we are a developing country where due to low resources/population ratio patients are either already anemic or with borderline hemoglobin levels and have to be transfused blood to build up hemoglobin. Because of the non-availability of fresh blood, usually stored blood is being transfused in our setup, which is deficient in platelets (short lifespan of platelets; 8-10 days). Therefore, we did not find a positive correlation between PLR with pain scores in our study.

In our study, it was observed that patients with day 3 moderate pain as compared to those with low pain could be mobilized significantly later (2.40 ± 0.49 vs. 2.03 ± 0.17 days) and had a significantly higher duration of hospital stay (11.83 ± 2.01 vs. 10.00 ± 0.00 days) and patients with day 5 moderate pain as compared to those with low pain could be mobilized significantly later (2.43 ± 0.50 vs. 2.15 ± 0.36 days) and had a significantly higher duration of hospital stay (11.83 ± 2.01 vs. 10.75 ± 1.57 days).

The present study has the following limitations. The study is an observational cohort study, and may not necessarily reflect the real-world association of the studied biomarkers.

Substantial heterogenicity of our study population (wider range of age, i.e. 18-90 years) might have confounded our results as the immune response to surgical stress is different in different age groups. Spinal anesthesia alone was not adequate to provide surgical anesthesia to patients with intraoperative difficulties. Laboratory blood tests were not performed in the same laboratory, so the results may not be equivocal. Communication with older patients was difficult; therefore, VAS scores may not have exactly reflected the pain.

Recommendations

Multicenter studies in different ages, diseases, and anesthesia specifics should be conducted to validate the result. There should be long-term follow-up for the study. Laboratory tests should be conducted in one laboratory.

## Conclusions

The present study demonstrated a significant correlation between preoperative and postoperative NLR with pain after TJA. The correlation of CRP levels at different time intervals did not show a significant correlation with Day 3 and Day 5 pain scores. PLR at Pre-op., Day 3, and Day 5 did not show any significant correlation with post-operative pain at different time intervals. Post-operative pain is a significant component of the systemic stress response induced by surgical intervention. Integrating the NLR into clinical practice seems beneficial because it is easy, cheap, and convenient and can be calculated in daily practice without extra cost. In patients with age >60 years, history of myocardial infarction, TIA and anemia, the high NLR on admission can be a predictor of poorer postoperative outcomes including high inflammatory response/infection. This simple and inexpensive biomarker could be used for risk stratification and individualized perioperative pain management. Anesthetists can make decisions about the time and type of analgesic to be used based on NLR values. NLR can be used especially in geriatric patients who might have various central nervous system disorders like Alzheimer’s disease, dementia, age-related cerebral atrophy, Parkinsonism, etc. where expression of pain may be difficult, hence NLR can be used to provide maximum postoperative pain control and to facilitate early rehabilitation and prevention of postoperative complications.
